# Plasma Membrane Targeting of Protocadherin 15 Is Regulated by the Golgi-Associated Chaperone Protein PIST

**DOI:** 10.1155/2016/8580675

**Published:** 2016-10-27

**Authors:** Hongyun Nie, Yueyue Liu, Xiaolei Yin, Huiren Cao, Yanfei Wang, Wei Xiong, Yushuang Lin, Zhigang Xu

**Affiliations:** ^1^Shandong Provincial Key Laboratory of Animal Cells and Developmental Biology, Shandong University School of Life Sciences, Jinan, Shandong 250100, China; ^2^Taishan Medical University, Tai'an, Shandong 271000, China; ^3^School of Life Sciences, Tsinghua University, Beijing 100084, China

## Abstract

Protocadherin 15 (PCDH15) is a core component of hair cell tip-links and crucial for proper function of inner ear hair cells. Mutations of* PCDH15* gene cause syndromic and nonsyndromic hearing loss. At present, the regulatory mechanisms responsible for the intracellular transportation of PCDH15 largely remain unknown. Here we show that PIST, a Golgi-associated, PDZ domain-containing protein, interacts with PCDH15. The interaction is mediated by the PDZ domain of PIST and the C-terminal PDZ domain-binding interface (PBI) of PCDH15. Through this interaction, PIST retains PCDH15 in the trans-Golgi network (TGN) and reduces the membrane expression of PCDH15. We have previously showed that PIST regulates the membrane expression of another tip-link component, cadherin 23 (CDH23). Taken together, our finding suggests that PIST regulates the intracellular trafficking and membrane targeting of the tip-link proteins CDH23 and PCDH15.

## 1. Introduction

Inner ear hair cells are responsible for mechanoelectrical transduction (MET) that converts mechanical stimuli into electrical signals. MET occurs within stereocilia, the F-actin-based, and microvilli-like protrusions on the apical surface of hair cells [1]. Stereocilia are organized into several rows of increasing heights, forming a staircase-like structure. Several types of extracellular links, including tip-links, top-connectors, lateral-links, ankle-links, and kinociliary-links, connect stereocilia with each other and with the microtubule-based kinocilium [2]. Among these extracellular links, tip-links are of great importance. Tip-links connect the tip of each stereocilium to the side of its taller neighboring stereocilium and are directly involved in the MET process [3]. The yet unidentified MET channels localize at the lower end of tip-links and form the so-called MET machinery together with the lower end of tip-links as well as other proteins [4]. When stereocilia are deflected in the excitatory direction, the tension of tip-links increases and the open probability of MET channels also increases, resulting in the influx of cations into hair cells [1].

Protocadherin 15 (PCDH15) is an atypical cadherin that contains eleven extracellular cadherin (EC) repeats, whereas classical cadherins usually have only five EC repeats. Genetic, biochemical, immunochemical, and structural evidence indicated that PCDH15 and cadherin 23 (CDH23), another atypical cadherin, form the lower and higher part of tip-links, respectively [5–7]. PCDH15 and CDH23 interact with each other via the most N-terminal two EC repeats and create a ~170 nm long extracellular link under endolymph-like calcium levels [7–9]. Mutations of* PCDH15* gene are responsible for syndromic hearing loss Usher 1F or nonsyndromic hearing loss DFNB23 [10–12].* Pcdh15* mutations are also responsible for the hearing and balancing deficits of Ames waltzer (av) mice [13, 14]. In av mice, stereociliary tip-links are reduced or even absent, depending on the nature of the mutations, and hair cells are degenerated eventually, resulting in deafness and vestibular dysfunction [15].

**Figure 1:**
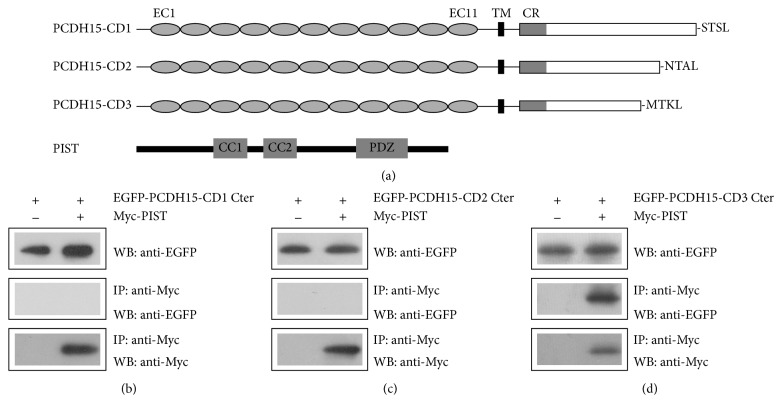
PCDH15-CD3 interacts with PIST. (a) Schematic diagram of PCDH15 and PIST domain structure. EC, extracellular cadherin repeats; TM, transmembrane domain; CR, common region; CC1, coiled-coil domain 1; CC2, coiled-coil domain 2; PDZ, PSD-95/discs large/ZO-1 domain. The C-terminal PBI of PCDH15 isoforms (STSL, NTAL, and MTKL) is also indicated. (b) Western blots showing coimmunoprecipitation (co-IP) of EGFP-tagged mouse PCDH15-CD1 cytoplasmic domain with Myc-tagged human PIST. (c) Western blots showing co-IP of EGFP-tagged mouse PCDH15-CD2 cytoplasmic domain with Myc-tagged human PIST. (d) Western blots showing co-IP of EGFP-tagged mouse PCDH15-CD3 cytoplasmic domain with Myc-tagged human PIST. IP indicates antibody used for immunoprecipitation and WB indicates antibody used for detection.

Three prominent PCDH15 isoforms are generated by alternative pre-mRNA splicing, which are PCDH15-CD1, PCDH15-CD2, and PCDH15-CD3 [6]. These PCDH15 isoforms differ in their cytoplasmic domains (Figure 1(a)) and show different spatiotemporal expression pattern in the developing and mature inner ear [6]. Noticeably, different PCDH15 isoforms contain different PDZ binding interfaces (PBI) at their C-termini, which consist of the last four amino acids and are responsible for binding to PDZ domains. Different PCDH15 isoforms function redundantly during hair cell development, whereas PCDH15-CD2 was shown to be essential for tip-links in mature auditory hair cells [16, 17]. All PCDH15 isoforms could interact with TMHS (also called LHFPL5), an integral component of MET machinery of cochlear hair cells [18]. PCDH15-CD2, but not PCDH15-CD1 or PCDH15-CD3, also binds TMIE, another MET machinery component [19]. Furthermore, all PCDH15 isoforms directly interact with TMC1 and TMC2, the candidate MET channels, and core component of MET machinery [20, 21]. These data suggest that PCDH15 is an important component of the MET machinery.

Several other PCDH15-binding proteins have also been identified so far. PCDH15-CD1 was shown to interact with PDZ domain-containing protein harmonin via its C-terminal PBI [22, 23]. Further investigation showed that the stereociliary localization of PCDH15 is affected in* harmonin* knockout mice [24]. PCDH15-CD1 also interacts with Myosin VIIA, and the stereociliary localization of PCDH15 is perturbed in* myosin VIIA* mutant mice [25]. Recently, PCDH15-CD2 was shown to interact with Myosin 3A, which might regulate the transportation of PCDH15 to stereociliary tips [26]. In the present work, we show that PCDH15-CD3, but not PCDH15-CD1 or PCDH15-CD2, interacts with PDZ domain-containing protein PIST, which might play an important role in the intracellular trafficking and plasma membrane targeting of PCDH15-CD3.

## 2. Materials and Methods

### 2.1. DNA Constructs and Antibodies

Chicken* Pcdh15-CD3 *cDNA was inserted into pBD-GAL4 Cam vector (Stratagene) to express the C-terminal 106 amino acids of chicken PCDH15-CD3 as bait protein. Mouse* Pcdh15* cDNAs were inserted into pcDNA3.1(+) to express full length PCDH15 isoforms with a Myc tag between the N-terminal signal peptide and the first EC repeat. Mouse* Pcdh15* cDNAs were inserted into pEGFP-C2 or modified pEGFP-C2 (EGFP replaced with Myc) to express EGFP-tagged or Myc-tagged PCDH15 cytoplasmic domains. The cDNA encoding mouse PCDH15-CD3 missing the last 4 aa (MTKL) at the C-terminus was inserted into the same vectors to express Myc-PCDH15-CD3 (-MTKL) and Myc-PCDH15-CD3 Cter (-MTKL). Human* PIST* cDNA was inserted into pEGFP-C2 or modified pEGFP-C2 to express full length PIST, PIST CC2-plus domain (146-274aa), and PIST PDZ domain (276-366aa) as EGFP-fusion or Myc-fusion proteins. Mouse monoclonal anti-EGFP antibody (Cat. number M20004) was from Abmart. Mouse monoclonal anti-Myc antibody (Cat. number M4439) was from Sigma-Aldrich.

### 2.2. Yeast Two-Hybrid Screen

The yeast two-hybrid screen was performed as previously described [27, 28]. Briefly, yeast strain AH109 (Clontech) was sequentially transformed with the bait plasmid and a chicken cochlear cDNA library in the HybriZAP two-hybrid vector [29]. Totally 2.4 × 10^6^ transformants were selectively screened using* HIS3* (at the presence of 2.5 mM of 3-amino-1,2,4-triazole) as the primary reporter gene. The positive colonies were further examined using two more reporter genes* ADE2* and* lacZ*. The prey vectors in triple-positive yeast colonies were recovered and the sequence of cDNA inserts was determined by Sanger sequencing.

### 2.3. Coimmunoprecipitation (Co-IP)

HEK293T cells were transfected with the expression vectors using jetPRIME Transfection Agent (Polyplus, Cat. number PT-114-15) according to the manufacturer's instructions. Transfected cells were washed with phosphate-buffered saline (PBS) 24 hours after transfection and lysed in ice-cold lysis buffer consisting of 150 mM NaCl, 50 mM Tris at pH 7.5, 1% (vol/vol) Triton X-100, 1 mM PMSF, and 1 × protease inhibitor cocktail (Roche). After centrifugation at 4°C, the supernatant was collected and incubated with immobilized anti-Myc antibody (Sigma-Aldrich, Cat. number E6654) at 4°C overnight. After washing five times with washing buffer (a modified lysis buffer containing 500 mM NaCl instead of 150 mM), the immunoprecipitated proteins were separated by polyacrylamide gel electrophoresis (PAGE) and then transferred to PVDF membrane. The blot was incubated with corresponding primary antibodies, followed by incubation with secondary antibodies (Bio-Rad), and the signals were detected with the ECL system (Cell Signaling Technology).

### 2.4. Immunofluorescence

COS-7 cells were grown on Gelatin-coated glass cover slips and transfected with the expression vectors. Transfected cells were fixed with 4% paraformaldehyde (PFA) in PBS for 15 minutes and then permeabilized and blocked with PBT1 (0.1% Triton X-100, 1% BSA, 5% heat-inactivated goat serum in PBS, pH 7.3) for 30 minutes, followed by incubation with primary antibody diluted in PBT1 over night at 4°C. After washing twice with PBT1 for 10 minutes and twice with PBT2 (0.1% Triton X-100, 0.1% BSA in PBS) for 5 minutes, cells were incubated with fluorescence-conjugated secondary antibody (Life Tech) in PBT2 for 1 hour, followed by two 5-minute PBT2 washes and two 5-minute PBS washes. For nuclei staining, cells were incubated with DAPI (Gen-View Scientific Inc.) for 15 minutes, followed by four 5-minute PBS washes and then mounted in Glycerol/PBS (1 : 1). The cells were imaged with a confocal microscope (LSM 700, Zeiss).

## 3. Results

To identify proteins that interact with PCDH15, we performed yeast two-hybrid screens of a chicken cochlear cDNA library using PCDH15 as bait.* HIS3* gene was used as the primary reporter gene for the screen in the presence of 3-amino-1,2,4-triazole (3-AT) that inhibits the autoactivation of* HIS3* reporter gene. When the intact cytoplasmic domain of chicken PCDH15-CD3 was used as bait, it autoactivated the* HIS3* reporter gene at the highest 3-AT concentration (15 mM) tested. Then various regions within the PCDH15-CD3 cytoplasmic domain were tested for autoactivation. The results showed that the fragment containing the C-terminal 106 amino acids does not activate* HIS3* reporter gene at the presence of 2.5 mM 3-AT, and this fragment was used in the following yeast two-hybrid screen. Among the positive clones that activate all the three reporter genes (*HIS3*,* ADE2*, and l*acZ*), several clones encode the PDZ-containing, Golgi-associated chaperone protein PIST. PIST has been shown to play important roles in regulating the membrane targeting of several transmembrane proteins such as frizzled, somatostatin receptor subtype 5 (SSTR5) and *β*1-adrenergic receptor (*β*1AR) [30–32].

We then performed coimmunoprecipitation (co-IP) experiments to verify the interaction between PCDH15 and PIST. From here on in the rest of our investigation, we focused on mammalian proteins. Our data showed that when overexpressed in HEK293T cells, EGFP-tagged cytoplasmic domain of mouse PCDH15-CD3 was co-IPed with Myc-tagged human PIST, whereas PCDH15-CD1 and PCDH15-CD2 were not (Figures 1(b)–1(d)). This result suggests that PCDH15-CD3, but not PCDH15-CD1 or PCDH15-CD2, interacts with PIST.

**Figure 2:**
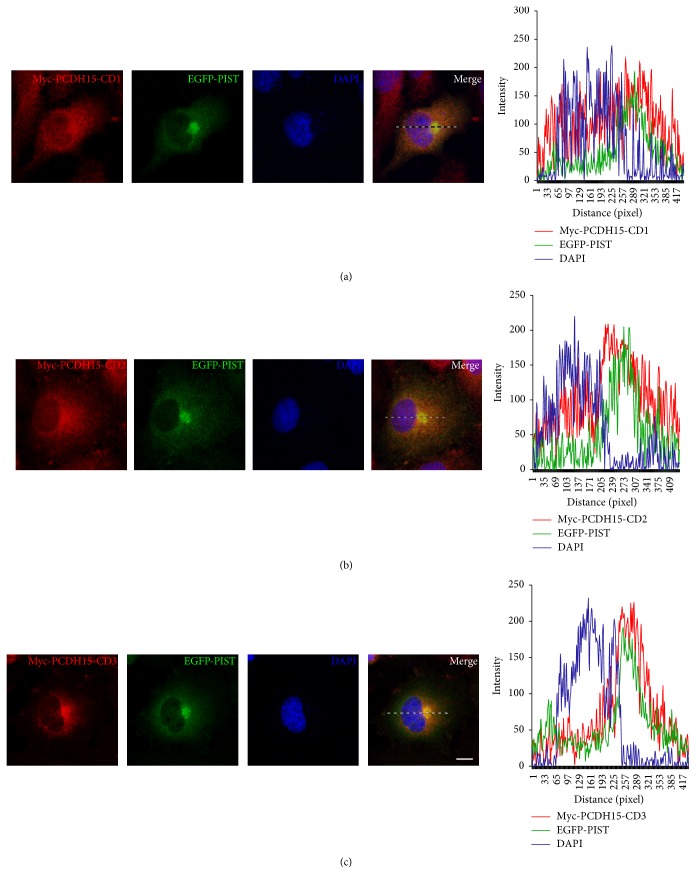
Colocalization of PCDH15-CD3 with PIST in COS-7 cells. (a) In the presence of EGFP-PIST, Myc-PCDH15-CD1 localizes in the cytoplasm as well as on the cell membrane. (b) In the presence of EGFP-PIST, Myc-PCDH15-CD2 localizes in the cytoplasm as well as on the cell membrane. (c) In the presence of EGFP-PIST, Myc-PCDH15-CD3 colocalizes with EGFP-PIST in the TGN. Myc-PCDH15 was stained with mouse anti-Myc antibody and then TRITC-conjugated goat anti-mouse secondary antibody. Nuclei were stained with DAPI. The fluorescent intensity was quantified using Image J. Scale bars, 10 *μ*m.

Previously we have shown that PIST binds to cadherin 23 (CDH23), the upper tip-link component, and retains CDH23 in the trans-Golgi network (TGN) in cultured cells [33]. To test whether this also applies to PCDH15, we analyzed the subcellular distribution of PCDH15 and PIST in cultured cells by immunofluorescence and confocal microscopy. The results showed that when coexpressed in COS-7 cells, EGFP-PIST mainly localizes in the TGN, whereas Myc-PCDH15-CD1 or Myc-PCDH15-CD2 localizes in the cytoplasm as well as on the plasma membrane, consistent with the fact that PCDH15-CD1 or PCDH15-CD2 does not interact with PIST (Figures 2(a) and 2(b)). In contrast, when cotransfected with EGFP-PIST and Myc-PCDH15-CD3, most (70.24%) cells show colocalization of PCDH15-CD3 with PIST in the TGN (Figure 2(c)).

**Figure 3:**
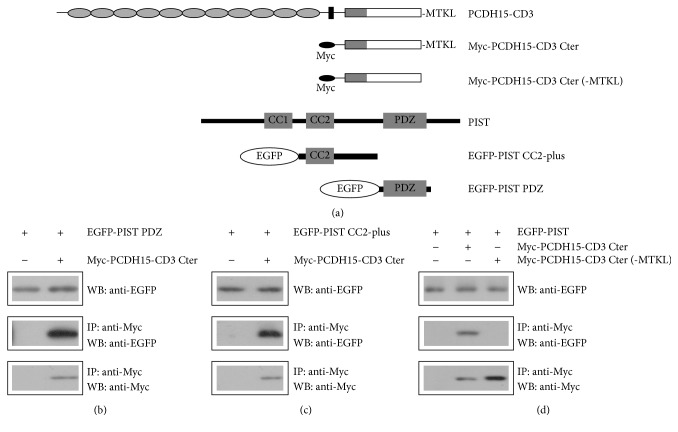
The C-terminal PBI of PCDH15-CD3 is necessary for the interaction with PIST. (a) Schematic diagram of PCDH15 and PIST domains used in this experiment. (b) Western blots showing coimmunoprecipitation (co-IP) of EGFP-tagged human PIST PDZ domain with Myc-tagged mouse PCDH15-CD3 cytoplasmic domain. (c) Western blots showing co-IP of EGFP-tagged human PIST CC2-plus region with Myc-tagged mouse PCDH15-CD3 cytoplasmic domain. (d) Western blots showing co-IP of EGFP-tagged human PIST with Myc-tagged mouse PCDH15-CD3 cytoplasmic domain with or without the C-terminal PBI (MTKL). IP indicates antibody used for immunoprecipitation and WB indicates antibody used for detection.

**Figure 4:**
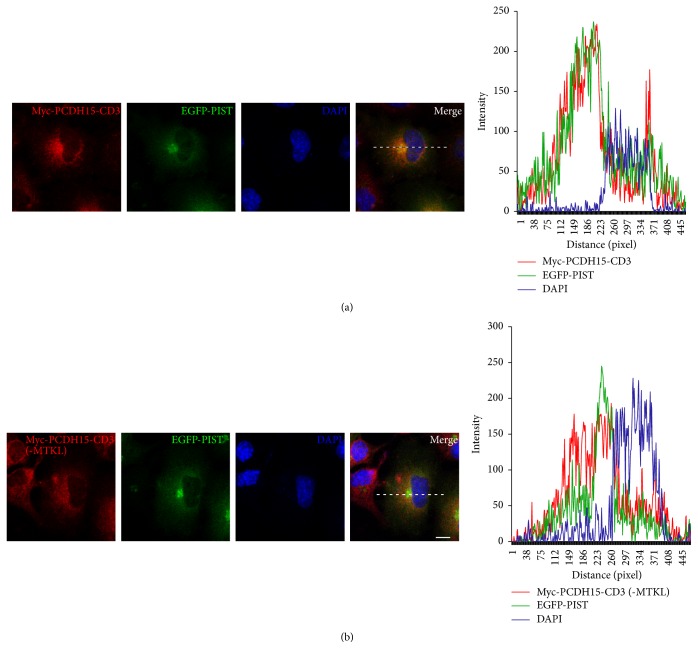
The C-terminal PBI of PCDH15-CD3 is necessary for its retention in the TGN of COS-7 cells by PIST. (a) In the presence of EGFP-PIST, Myc-PCDH15-CD3 colocalizes with EGFP-PIST in the TGN. (b) In the presence of EGFP-PIST, Myc-PCDH15-CD3 lacking the C-terminal PBI (MTKL) localizes in the cytoplasm as well as on the cell membrane. Myc-PCDH15 was stained with mouse anti-Myc antibody and then TRITC-conjugated goat anti-mouse secondary antibody. Nuclei were stained with DAPI. The fluorescent intensity was quantified using Image J. Scale bars, 10 *μ*m.

We then performed co-IP experiments to explore which regions/motifs in PCDH15-CD3 and PIST are responsible for the interaction between them. PIST contains a PDZ domain near the C-terminus and two coiled-coil (CC) domains in the middle part (Figure 3(a)). Co-IP experiments with EGFP-tagged PIST PDZ domain (276-366aa) or the second CC domain (CC2) plus the amino acids between CC2 and PDZ domain (CC2-plus, 146-274aa) revealed that both domains could be co-IPed with Myc-PCDH15-CD3 cytoplasmic domain (Figures 3(b) and 3(c)). PCDH15-CD1, PCDH15-CD2, and PCDH15-CD3 have unique C-termini (STSL, NTAL, and MTKL, resp.), which belong to type I PBI [34]. We found that when the C-terminal MTKL of PCDH15-CD3 was removed, EGFP-PIST could not be co-IPed with Myc-tagged PCDH15-CD3 cytoplasmic domain anymore (Figure 3(d)). In line with this, Myc-tagged PCDH15-CD3 lacking the C-terminal MTKL is not retained in the TGN by EGFP-PIST anymore (Figures 4(a) and 4(b)). These data suggest that the principal interaction between PCDH15-CD3 and PIST utilizes the PDZ binding interface.

**Figure 5:**
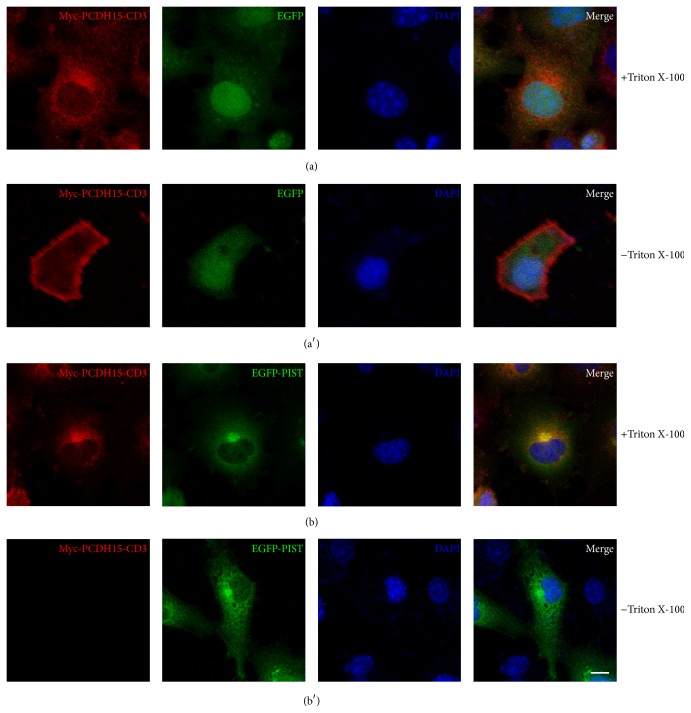
PIST reduces the membrane expression of PCDH15-CD3 in COS-7 cells. (a) Myc-PCDH15-CD3 immunoreactivity shows cytoplasmic and plasma membrane localization when coexpressed with EGFP. (a′) Myc-PCDH15-CD3 immunoreactivity using a nonpermeable protocol shows clear plasma membrane localization when coexpressed with EGFP. (b) Myc-PCDH15-CD3 immunoreactivity colocalizes with EGFP-PIST in the TGN. (b′) The plasma membrane localization of Myc-PCDH15-CD3 immunoreactivity examined using a nonpermeable protocol disappears when EGFP-PIST is present. For the nonpermeable protocol, staining was performed as normal except that Triton X-100 was excluded. Myc-PCDH15 was stained with mouse anti-Myc antibody and then TRITC-conjugated goat anti-mouse secondary antibody. Nuclei were stained with DAPI. Scale bar, 10 *μ*m.

Finally, we examined whether the association with PIST affects the membrane targeting of PCDH15-CD3. When coexpressed with EGFP, Myc-PCDH15-CD3 mainly localizes in the cytoplasm and on the plasma membrane (Figure 5(a)). The plasma membrane localization of Myc-PCDH15-CD3 could be observed more clearly when the extracellular Myc epitope was labeled by anti-Myc antibody without cell permeabilization (Figure 5(a′)). In contrast, when EGFP-PIST is present, Myc-PCDH15-CD3 colocalizes with EGFP-PIST in the TGN, and the membrane labelling in nonpermeabilized cells disappeared completely (Figures 5(b) and 5(b′)). These data suggest that PIST retains PCDH15-CD3 in the TGN and reduces the membrane expression of PCDH15-CD3.

## 4. Discussion

PCDH15 and CDH23 form the lower and upper part of tip-links, respectively, and are indispensable for hearing transduction. Besides tip-links, PCDH15 and CDH23 also localize at kinociliary-links, transient lateral-links, and ribbon synapse of hair cells [6, 7, 35–37]. It has been suggested that the transportation of PCDH15 to apical (stereociliary bundle) and basal (ribbon synapses) poles depends on distinct trafficking mechanisms. Apically transported PCDH15 was shown to associate with the Arf1-positive early endosomal vesicles and colocalize with the early endosomal marker Rab5, whereas basally transported PCDH15 was shown to associate with AP-1-positive post-trans-Golgi vesicles [38]. The detailed mechanism of PCDH15 transportation remains elusive.

In the present work we show that PIST might play an important role in regulating the intracellular traffic and membrane targeting of PCDH15. Previously we have shown that PIST regulates the membrane expression of CDH23 [33]. Taken together, our work suggests that PIST regulates the intracellular transportation of both tip-link components, PCDH15 and CDH23. PIST is a PDZ-containing, Golgi-associated chaperone protein, also named as FIG, GOPC, or CAL. PIST plays important roles in regulating the membrane targeting of transmembrane proteins [30–32]. Notably, PIST was shown to colocalize with the early endosome marker Rab5 and the TGN/endosome marker Rab14 [39]. Our data reveal that PCDH15-CD3 and CDH23 bind PIST via their C-terminal PBI and colocalize with PIST in the TGN, which might regulate the transportation of PCDH15 and CDH23 to the apical surface of hair cells.

Interestingly, we found that, besides the PDZ domain, the CC2 domain and its downstream amino acids (CC2-plus) of PIST also interact with PCDH15-CD3. This was also observed in the interaction between PIST and CDH23 [33]. Nevertheless, it seems that PDZ/PBI mediate the principal interaction between PIST and PCDH15-CD3 or CDH23 because deletion of the C-terminal PBI abolishes the interaction completely. PCDH15-CD1, PCDH15-CD2, and PCDH15-CD3 all contain a type I PBI, but only PCDH15-CD3 binds PIST and colocalizes with PIST in the TGN. Previously, PCDH15-CD1 has been shown to interact with PDZ domain-containing protein harmonin [22, 23]. The different C-terminal PBI of the three PCDH15 isoforms might mediate interaction with different PDZ domain-containing proteins and render the PCDH15 isoforms different function or regulation.
